# A Comparative Study of the Effect of Sutureless Versus Multiple Sutures Technique on Complications following Third Molar Surgery in Nepalese Subpopulation

**DOI:** 10.1155/2020/9314762

**Published:** 2020-02-11

**Authors:** Arun Kr. Mahat, Ram Yadav, Anjani Kr. Yadav, Pradeep Acharya, Ashok Dongol, Alok Sagtani, Mehul R. Jaisani

**Affiliations:** ^1^Department of Oral and Maxillofacial Surgery, B. P. Koirala Institute of Health Sciences, Dharan, Nepal; ^2^Department of Oral and Maxillofacial Surgery, Kathmandu Medical College, Kathmandu, Nepal

## Abstract

**Objective:**

To compare the effect of sutureless versus multiple sutures technique on postoperative variables such as pain, swelling, and trismus after surgical removal of the third molar in Nepalese subpopulation. *Materials and Methods*. Forty-eight patients were equally grouped into multiple sutures (group 1) and sutureless (group 2) groups using a computer-generated random table. The same operator performed all the surgical procedures. Postoperative variables such as pain, swelling, and trismus were measured by a single concealed observer using a 10 cm Numerical Rating Scale (NRS), flexible plastic measuring tape, and vernier caliper preoperatively and on 1^st^, 2^nd^, and 7^th^ postoperative days.

**Results:**

This study showed significantly more swelling measured from the gonion to lateral canthus in group 1 than in group 2 on all postoperative periods (*P* < 0.05). The mean NRS score was significantly higher in group 1 on the 1^st^ postoperative day (*P* < 0.05). The mean NRS score was significantly higher in group 1 on the 1^st^ postoperative day (

**Conclusion:**

Our results support the use of sutureless technique after third molar surgery to minimize postoperative morbidity and the overall operative time and reduce the cost within the Nepalese subpopulation.

## 1. Introduction

Impacted tooth is defined as a tooth that is prevented from erupting into position [1] within the expected time [2] due to a physical barrier within the path of eruption [3]. Third molar impactions are associated with several acute or chronic pathological changes, such as pain, infection, caries, periodontal disease, root resorption, bone loss, cyst formation, and benign tumors, justifying its removal. Pain, swelling, decreased mouth opening, and temporary inability to work are the generally accepted inevitable postoperative consequences following surgical extraction. This is further associated with a significant deterioration in oral health-related quality of life in the immediate postoperative period [4] and an increase in the total expenditure for removal of third molars [5, 6]. The magnitude of these sequelae depends on the extent of inflammatory response resulting from the extent of tissue damage produced [7], which in turn depends on certain demographics including age, gender, oral health status, and anatomic and operative factors such as increased surgical difficulty, magnitude of ostectomy, and duration of surgery [7]. Different closure techniques with or without incorporation of drains [8]; use of drugs such as analgesics [9], corticosteroids [10], and antibiotics [11]; and physical therapeutic methods such as cryotherapy [12], soft laser application [13], and sutureless techniques [14–18] are among the number of modalities reported in the literature to minimize postoperative pain, swelling, and trismus. Damage to the capillary vessels and the release of inflammatory cytokines as a result of trauma lead to increased permeability of vessels and accumulation of serosanguineous fluid and exudate [16]. Suturing the flap back has an advantage of promoting healing by primary intention, convenient maintenance of effective oral hygiene, and hemorrhage control. However, suturing creates a one-way valve that allows food debris to enter the socket but does not allow to escape[19].This leads to local infection, inflammation, edema, clot necrosis, alveolar osteitis, and pain. Sutureless closure with minimal manipulation of soft tissues and healing by secondary intention, decreasing time of surgery, leaving a self-drainage pathway for inflammatory exudate, and thereby reducing postoperative inflammation with impact on improving the oral health related quality of life index. Abundant data exists regarding the advantages of sutureless technique after third molar surgery like less pain, swelling, and trismus with comparatively few undesirable effects [5, 7, 15–18] related to healing by secondary intention. In addition, there may be high potential for the formation of a periodontal pocket in relation to the adjacent second molar, but however, the literature is equivocal. To the best of our knowledge, no study has been reported for our population with completely different oral hygiene status. Furthermore, there are only few of the comparative studies done between the newer sutureless technique and the conventional method of closure after third molar surgery. Thus, we compared the effects of multiple sutures and sutureless technique in patients referred for third molar surgical extraction in BPKIHS with the objective to evaluate the effectiveness of both techniques in reducing postoperative complications. This would help us to improve the outcome on oral health-related quality of life and decrease the treatment cost for our patients.

## 2. Materials and Methods

This was a prospective, experimental, randomized clinical trial on humans. Forty-eight healthy patients with a total or partial impacted mandibular third molar consenting to participate in the study were divided into multiple sutures (*n* = 24) and sutureless (*n* = 24) groups by the help of computer-generated random table.

Patients with known systemic disease, pregnant and lactating women, contraindication to the drugs or anaesthetic in the surgical protocol, and patients with pericoronitis currently under antibiotics within 1 week were excluded from the study.

Ethical approval was taken from the institutional ethical review board, BPKIHS (IERB/223/014). Consent was taken after giving detailed information regarding the research.

### 2.1. Surgical Protocol

The surgical procedures were performed by a single concealed consultant until the time of flap repositioning. The standard surgical procedure for extraction of the impacted mandibular third molar using Ward's incision was practiced. Patients were not given preoperative antimicrobial or other drugs (NSAIDs) that might influence healing and pain. In group 1 (multiple sutures), the flap was repositioned and sutured (3-0 silk suture, interrupted). In the sutureless group (group 2), the flap was repositioned and allowed to passively fall into a natural position, often leaving the socket slightly open. Sterile gauze was placed to obtund bleeding and stabilize the flap. The patients were allowed to recover for 20 to 30 minutes and then rechecked for flap position and hemorrhage. No dressing was applied to the open socket. The mean duration of surgery, from incision to suturing/flap repositioning, was recorded in minutes. Patients were given Cap. Amoxycillin 500 mg PO 8 hourly for 5 days and Tab. Ibuprofen 400 mg + Paracetamol 325 mg PO 8 hourly for 1 day then SOS, along with oral and written postoperative instructions. Sutures were removed after 7 days in group 1.

### 2.2. Evaluation Procedure

Patients were evaluated in a concealed manner by the same independent observer preoperatively and postoperatively on the first, second, and seventh days after surgery. Pain was evaluated using a 10 cm numeric rating scale (NRS) with Nepali manuscript. Trismus was evaluated by measuring the distance between the mesial-incisal corners of the upper and lower right central incisors at maximum mouth opening in mm, using vernier calipers. The facial swelling in cm was determined by measuring the distance from the corner of the mouth to the attachment of the earlobe following the bulge of the cheek and the distance from the outer canthus of the eye to the angle of the mandible using a plastic measuring tape. The difference between each postoperative and preoperative measurement indicated the trismus and facial swelling for that day. Postoperative bleeding was recorded if the patient reported to the department with the complaint.

### 2.3. Sample Size

We conducted the study considering swelling as a continuous variable on both independent suture and sutureless groups with 1 : 1 subject. Based on the previous study by O. D. Osunde, R. A. Adebola, B. D. Saheeb “A comparative study of the effect of sutureless and multiple suture techniques on inflammatory complications following third molar surgery” [17], the distribution was assumed to be normal with a standard deviation of 0.12. As the mean of true difference in the experimental group and control group was 0.1, we needed 24 subjects each in multiple sutures and sutureless group to reject the null hypothesis so that the population mean of sutureless and suture groups are equal with probability (power) as 0.8. Probability of type 1 error (*α*-error) associated with this test was 0.05.

### 2.4. Statistical Methods Employed

The data were entered in Microsoft Excel 2007 and analyzed using Statistical Package for Social Sciences (SPSS) version 11.5. For descriptive statistics, mean, standard deviation, and percent proportion were calculated and tabular presentations were made. Variables which did not follow normal distribution were analyzed through a nonparametric test, i.e., Mann–Whitney's *U* test. Normally distributed variables were analyzed by the independent sample *T*-test. Measurements of association of categorical variables were done through the chi-squared test and odds ratio was calculated with 95% confidence limit.

## 3. Results

**Figure 1:**
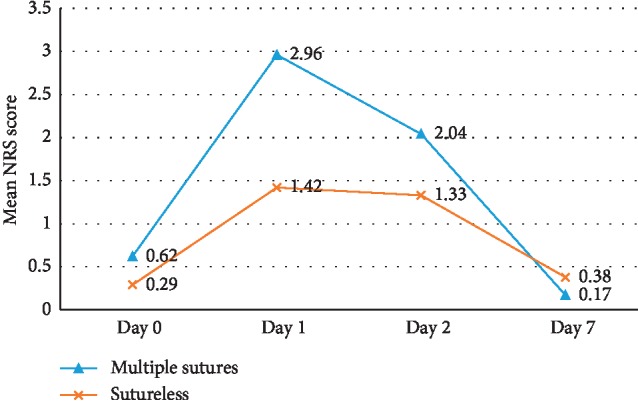
Comparison of pain between group 1 (multiple sutures) and group 2 (sutureless) on patients undergoing third molar surgery using NRS score.

**Figure 2:**
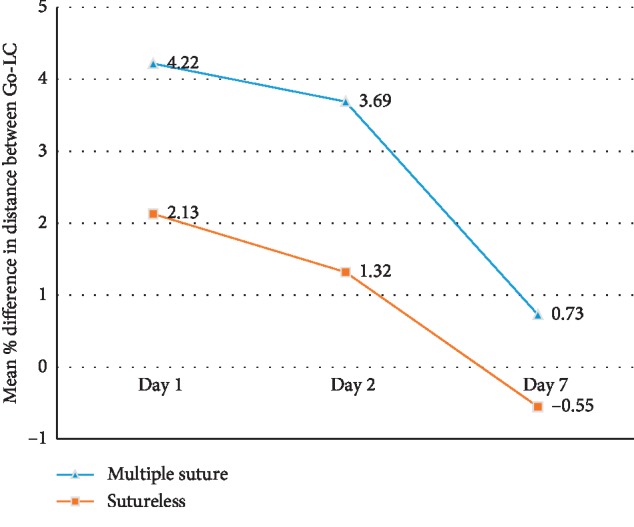
Comparison of swelling (gonion to lateral canthus) between group 1 (multiple sutures) and group 2 (sutureless) on patients undergoing third molar surgery.

**Figure 3:**
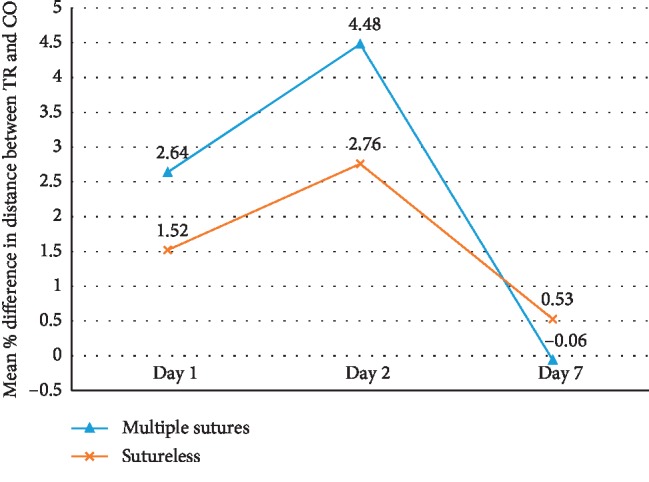
Comparison of swelling (tragus to commissure of lips) between group 1 (multiple sutures) and group 2 (sutureless) on patients undergoing third molar surgery.

**Figure 4:**
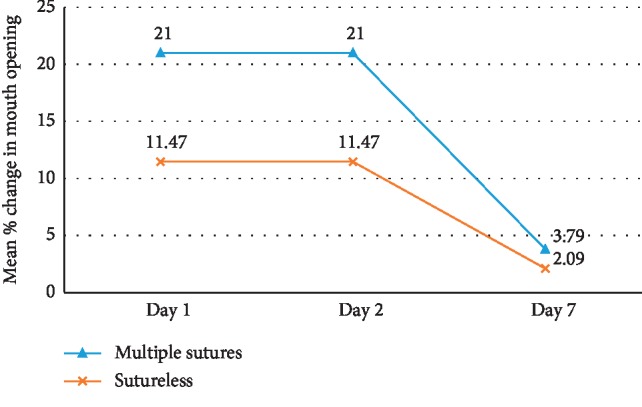
Comparison of trismus (mouth opening) between group 1 (multiple sutures) and group 2 (sutureless) on patients undergoing third molar surgery.

There were no significant differences in demographic, anatomic, operative, and baseline patient characteristics except for the number of local anaesthetic blocks which were more in the sutureless group (*P*=0.02) (Table 1). The result of the present study showed that swelling was significantly more in the multiple sutures group when measured from the gonion to lateral canthus on all postoperative days (*P*=0.002 − 0.004). Similarly, the mean NRS score was significantly higher in group 1 on the 1^st^ postoperative day (*P*=0.01). Though mean duration of surgery, swelling as measured from tragus to commissure, trismus, NRS score, total number of analgesics consumed, and complications were more in the multiple sutures group, the difference was not statistically significant (Figures 1–4). The mean number of analgesics intake was higher in group 1, except for the 5^th^ and 6^th^ postoperative day. An equal number of analgesics was consumed in the 5^th^ postoperative day. Though the number of analgesics consumed was greater in the second postoperative day in both groups, the frequency decreased till the 7^th^ day. Overall, there was no statistically significant difference in analgesic consumption between two groups. Complications like angular cheilitis, rashes, diarrhea, fever, erythema, and fainting were seen in a greater number of patients in group 1. However, there was no statistically significant association between occurrence of complications between the two groups. (*P*=0.33). Limitations of daily activities were only reported for the multiple sutures group.

## 4. Discussion

Third molar surgery is a routine clinical practice with a threefold increase in adverse effects on the quality of life in patients who experience pain, swelling, and trismus, compared to those who are asymptomatic [20]. Sutureless procedure is evolving as a simple and viable option for minimizing the postoperative morbidity, decreasing the overall time for the procedure and also reducing the financial burden associated with suture material cost and follow-up visit required for suture removal after third molar surgeries. The cost limiting impact of sutureless technique is also one of the influencing factors especially for patients from developing countries.

The present study compares the effect of sutureless (secondary closure) and multiple suture (primary closure) techniques on postoperative pain, swelling, and trismus. The mean number of sutures in the multiple sutures group was 1.92 ± 0.50 with a range of 1–3.

As pain is a subjective experience influenced by many factors such as the patient's age, cultural background, educational level, previous experience of pain, pain threshold and tolerance; the assessment of pain may be difficult. An 11-point pain assessment, Numerical Rating Scale (NRS) being more useful than Verbal Rating Scale (VRS) or the Visual Analogue Scale (VAS) [21–24] and total analgesics consumption after the prescribed dose, has been used in this study. Pain was significantly greater in the first postoperative day in the multiple sutures group (*P*=0.01). The difference was not statistically significant on other postoperative days, which is in accordance with the study by Rakprasitkul and Pairuchvej [25] which differs from most other studies [5, 15–18]. However, 8 patients in the multiple sutures group and 3 patients in the sutureless group had NRS score greater than 3 on the 1^st^ postoperative day and 3 patients in each group on the 2^nd^ postoperative day. Our study reported a decline in the mean number of analgesics consumed with each postoperative day in both groups, and the difference was not significant.

Swelling was measured using a flexible plastic measuring tape as described by Gabka and Matsumura [26] by measuring the distance from the corner of the mouth to the attachment of the earlobe following the bulge of the cheek and the distance from the outer canthus of the eye to the angle of the mandible [17]. Perhaps, more accurate methods such as ultrasonography [27], computerized tomography scanning, or magnetic resonance imaging exist for making precise measurements of facial soft tissue volume; using flexible plastic measuring tape is a simple, cost-effective, and time-saving method which provides numerical data for determination of soft tissue contour changes [17]. Also, a single observer concealed to the distribution of patient with fixed facial landmarks has little discrepancy when utilized to compare edema at different time intervals.

In our study, the mean percentage swelling of the patients when measured from the gonion to lateral canthus was maximum on the 1^st^ postoperative day which regressed on the 2^nd^ and 7^th^ postoperative days in both the groups but was significantly higher in the multiple sutures group. On the contrary, the mean percentage swelling when measured from the tragus to commissure of lips was higher on the 2^nd^ postoperative day as compared to the 1^st^ and 7^th^ postoperative days. The swelling was higher in the multiple sutures group, but the difference was not significant between the two groups. Though we could not find any article comparing the linear measurements separately, most literature [8, 14–18, 28, 29] studies agree to the fact that there is more postoperative swelling in the multiple sutures group.

With regard to trismus, interincisional distance is and has remained the most consistent and dependent measure over the years [10]. The mean percentage for trismus was found to be more in the multiple sutures group on all postoperative days with maximum trismus present on the 2^nd^ postoperative period followed by the 1^st^ and 7^th^ postoperative days similar to many studies [8, 14–18, 28, 29]; however, the difference was not significant.

In our study, complications were seen in 8 patients in the multiple sutures group and 5 patients in the sutureless group. We reported angular cheilitis in 10 patients to be the most common complication with one patient each with erythematous swelling on the right neck up to the clavicle with increase in local temperature on the second postoperative day; fainting on the operative day; diarrhea and rashes on the 3^rd^ day; and fever on the 2^nd^ day. The occurrence of angular cheilitis can be explained by the need for retraction of cheek during the procedure. Rashes and diarrhea could be due to the possible side effect of the antibiotics, but the patient did not report to the department immediately. So, no modification in postoperative antibiotic use was done. However, fever and erythema could be attributed to the inflammatory reactions and we could not find any literature reporting these complications. All the complications, except angular cheilitis, were reported in the multiple sutures group. We did not have any cases with bleeding and dry socket contrary to the reports in other literature studies [30] which can be explained by the fact that we gave both the written and verbal postextraction instructions and the operator made sure that it was understood by the patient. The instructions were reinforced in the follow-up visits.

The limitation of daily activities was said to be present if patients were unable to work after third molar surgery [4]. This was recorded in all postoperative visits. We saw limitations of daily activities only in the multiple sutures group with a mean duration of 0.29 ± 0.75 days (maximum = 3 days). This is a limiting factor for the conventional technique of closure after third molar surgery as it greatly influences the financial burden.

Some studies have reported that there may be high potential for the formation of a periodontal pocket in relation to the adjacent second molar with sutureless closure [17]. However, studies by Magnus et al. [31] and Woolf et al. [32] and a recent meta-analysis [33] concluded that there are no significant differences on the outcome between complete and partial wound closure and also referred that the available studies are heterogeneous and do not produce high level of scientific evidence. Apparently, the flap design and suture technique even with an exposed area distal to the second molar did not result in a periodontal defect if properly carried out [18].

The limitations of this study are short follow-up, experienced surgeon, and possibility of local anaesthetic blocks acting as a confounding factor. Comparison of the delayed presentation complications if any among the two groups was not possible due to short follow-up duration of the study. Similarly, the study was conducted within the Nepalese subpopulation. Thus, multicentric trials would be recommended globally to generalize the results of the present study.

Thus we recommend to reassess our practice in third molar surgery and consider sutureless technique as a cost-effective alternative to reduce postoperative morbidity of third molar surgery specially in developing countries like Nepal. We also suggest to perform a multicentric long-term study involving general dentists and patients to find out long-term complications and reproducibility of the result in routine practices.

## Figures and Tables

**Table 1:** Demographic, anatomic, operative, and baseline patient characteristics.

Characteristics	Multiple suture	Sutureless	*P* value
Age			
Mean ± SD	28.63 ± 8.75	28.38 ± 6.79	0.91
Range	18–52	18–46	
Gender			
Male	16	12	0.24
Female	8	12	
Tooth			
48	13	11	0.56
38	11	13	
Impaction			
Mesioangular	14	15	1
Horizontal	4	3	
Vertical	2	2	
Distoangular	4	4	
Impaction			
Class I	8	11	0.37
Class II	13	13	
Class III	3	0	
Impaction			
Position A	15	15	1
Position B	9	8	
Position C	0	1	
Number of blocks (LA) required	1.29 ± 0.46	1.04 ± 0.20	**0.02**
Duration of surgery (minutes)	28.50 ± 19.31	24.21 ± 11.09	0.60
Guttering			
No	4	2	0.67
Yes	20	22	
Sectioning			
No	12	9	0.38
Yes	12	15	
Numbers of sutures placed	1.92 ± 0.50	0.00	Not valid
Swelling			
Before GO_LC	10.92 ± 0.52	10.72 ± 0.57	0.20
Before TR_CO	11.73 ± 0.61	11.37 ± 0.82	0.09
Trismus			
Before MO	43.89 ± 3.96	46.52 ± 5.97	0.08
NRS 0	0.62 ± 1.74	0.29 ± 0.55	0.64

## Data Availability

Data regarding the research can be made available when requested.
